# GRIM-19 Disrupts E6/E6AP Complex to Rescue p53 and Induce Apoptosis in Cervical Cancers

**DOI:** 10.1371/journal.pone.0022065

**Published:** 2011-07-12

**Authors:** Ying Zhou, Ying Wei, Jing Zhu, Qingyuan Wang, Liang Bao, Yang Ma, Yu Chen, Dingqing Feng, Aijin Zhang, Jie Sun, Shreeram C. Nallar, Keng Shen, Dhananjaya V. Kalvakolanu, Weihua Xiao, Bin Ling

**Affiliations:** 1 Department of Obstetrics and Gynecology, Anhui Provincial Hospital Affiliated to Anhui Medical University, Hefei, Anhui, The People's Republic of China; 2 Hefei National Laboratory for Physical Sciences at Microscale and School of Life Sciences, University of Science and Technology of China, Hefei, Anhui, The People's Republic of China; 3 Department of Microbiology and Immunology, Greenebaum Cancer Center, University of Maryland School of Medicine, Baltimore, Maryland, United States of America; 4 Department of Obstetrics and Gynecology, Peking Union Medical College Hospital, Beijing, The People's Republic of China; Cornell University, United States of America

## Abstract

**Background:**

Our previous studies showed a down-regulation of GRIM-19 in primary human cervical cancers, and restoration of GRIM-19 induced tumor regression. The induction of tumor suppressor protein p53 ubiquitination and degradation by E6 oncoportein of high risk-HPV through forming a stable complex with E6AP is considered as a critical mechanism for cervical tumor development. The aims of this study were to determine the potential role of GRIM-19 in rescuing p53 protein and inducing cervical cancer cell apoptosis.

**Methodology/Principal Findings:**

The protein levels of GRIM-19 and p53 were detected in normal cervical tissues from 45 patients who underwent hysterectomy for reasons other than neoplasias of either the cervix or endometrium, and cervical cancer tissues from 60 patients with non-metastatic squamous epithelial carcinomas. Coimmunoprecipitation and GST pull-down assay were performed to examine the interaction of GRIM-19 with 18E6 and E6AP *in vivo and in vitro* respectively. The competition of 18E6 with E6AP in binding GRIM-19 by performing competition pull-down assays was designed to examine the disruption of E6/E6AP complex by GRIM-19. The augment of E6AP ubiquitination by GRIM-19 was detected in vivo and in vitro ubiquitination assay. The effects of GRIM-19-dependent p53 accumulation on cell proliferation, cell cycle, apoptosis were explored by MTT, flow cytometry and transmission electron microscopy respectively. The tumor suppression was detected by xenograft mouse model.

**Conclusion/Significance:**

The levels of GRIM-19 and p53 were concurrently down regulated in cervical cancers. The restoration of GRIM-19 can induce ubiquitination and degradation of E6AP, and disrupt the E6/E6AP complex through the interaction of N-terminus of GRIM-19 with both E6 and E6AP, which protected p53 from degradation and promoted cell apoptosis. Tumor xenograft studies also revealed the suppression of p53 degradation in presence of GRIM-19. These data suggest that GRIM-19 can block E6/E6AP complex; and synergistically suppress cervical tumor growth with p53.

## Introduction

High-risk human papillomaviruses (HR-HPV), such as HPV18 and HPV16, is not only an important cause of cervical cancer [1], but also the pathogens of a subset of other tumors such as head and neck squamous carcinomas [2], lung cancer [3] upper aerodigestive tract cancer [4] and anogenital cancer [5]. The expression of viral oncoproteins E6 in HPV-positive cervical carcinomas [6] can interact with the E6-associated protein (E6AP) to form E6/E6AP complex that specifically induces the ubiquitination and rapid degradation of p53, nuclear transcription factor X-box binding 91 (NFX1-91) and PDZ domain-containing proteins through the proteasome pathway [7], [8], [9], [10]. p53 degradation is an essential requirement for the survival of HR-HPV-infected tumors; thus blocking E6/E6AP complex mediating p53 degradation may be an attractive approach for treating cancers with HR-HPV infection [11], [12], [13], [14].

GRIM-19 was originally identified as a tumor-suppressive protein that was involved in cell death [15] through the association and suppression of STAT3 [16], [17]; Its expression is down regulated in renal, prostate and cervical cancers [16], [17], [18], [19], [20]. Moreover, GRIM-19 suppresses oncogene-induced remodeling of cytoskeleton and cell motility [21]; and cell cycle progression by interacting with tumor suppressor p16Ink4a [22]. Thus, GRIM-19 exerts distinct mechanisms in a variety of cell types. Here we report that GRIM-19 induces p53 accumulation through a disruption of the E6/E6AP complex and an induction of auto-ubiquitination of E6AP in cervical cancer cells. This study demonstrates a novel function and a molecular mechanism by which GRIM-19 inhibits HR-HPV induced tumorigenesis by protecting p53 from degradation.

## Results

### GRIM-19 and p53 are concurrently downregulated in cervical cancers

**Figure 1:**
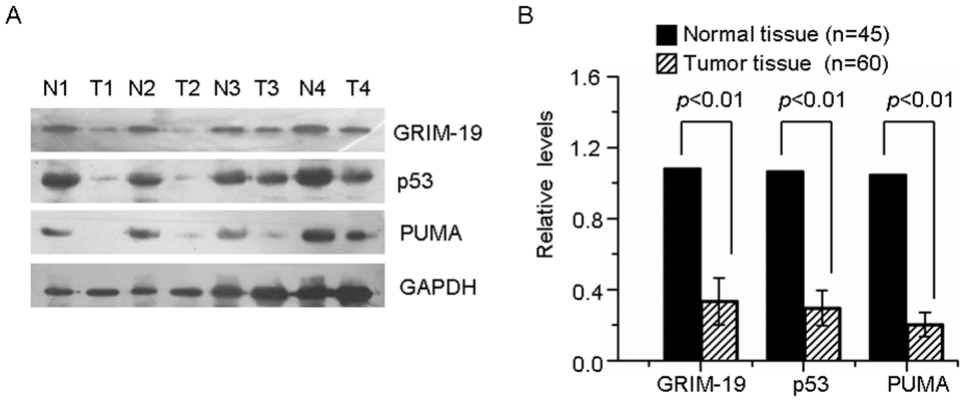
A correlation between the loss of GRIM-19 and p53 proteins in primary cervical cancers. (**A**) Total cellular extracts (50 µg) from primary cervical tumors (T) and normal cervical tissues (N) were examined for the expression of GRIM-19, p53 and PUMA by Western blotting. The representative results of Western blotting are shown. T1-T4 was from individual patients with cervical cancer. T1 and T2 were diagnosed as stage IIa; and T3 and T4 were diagnosed as stage Ia squamous epithelial carcinomas. (**B**) Quantitative analysis of the protein expression as measured by the optical density of each band. The ratio of the density from GRIM-19, p53, PUMA over the corresponding GAPDH (45 cases of normal tissue and 60 cases of tumor tissue) was calculated.

Our previously study demonstrated that GRIM-19 induces cervical tumor regression in a mouse xenograft model, suggesting a possible role of GRIM-19 in tumor growth regulation [20]. Since p53 tumor suppressor is also low expressed in cervical tumors, we further examined if there is a correlation between the levels of GRIM-19 and p53. The levels of GRIM-19 and p53 were significantly (*p*<0.01) lower in the tumors, and directly correlated with each other in >99% of cervical tumors (Fig. 1). Consistent with a low p53 in the tumors, a well-studied p53 target gene, PUMA, was also downregulated in the tumors compared to normal tissues (Fig. 1). These results demonstrated that GRIM-19 and p53 levels were concurrently suppressed, suggesting a potential link between GRIM-19 and p53 in cervical cancer.

### GRIM-19 augments p53 protein levels in cervical tumor cells

**Figure 2:**
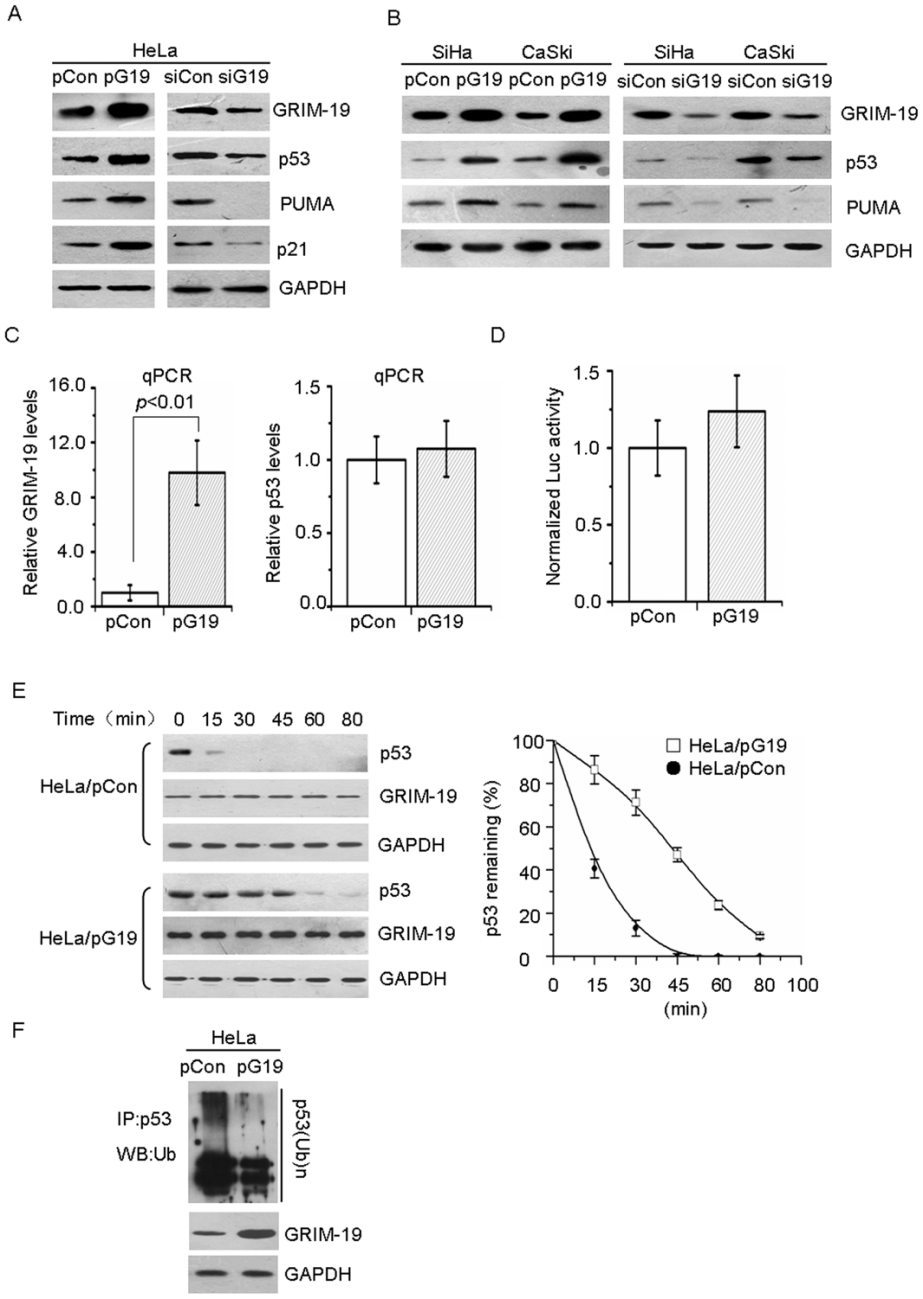
GRIM-19 induced p53 protein accumulation in HR-HPV infected cervical tumor cells. (**A & B**) The protein levels of GRIM-19 and p53 in primary cervical cancers. Western blotting with the indicated antibodies was performed using lysates from the indicated cell lines. (**C**) Effect of GRIM-19 on p53 mRNA expression. The mRNA of GRIM-19 (left panel) significantly high in HeLa/pG19 cells (* *p*<0.01), the right panel shows p53 mRNA expression. Differences are not statistically significant. (**D**) GRIM-19 did not affect the p53-promoter activity. A p53-Luc reporter was used. The data presented are the mean of three independent experiments with triplicate samples in each test. (**E**) GRIM-19 increases the half-life of p53. Cells were treated with CHX (100 µg/ml) for the indicated time periods and lysates were subjected for Western blotting with the indicated antibodies. Representative results from one out of three independent experiments are shown (left panel). The remaining value for p53 was calculated as the ratio of the densitometric values of p53 over GAPDH in each sample (right panel). The average remaining values from three independent experiments were plotted. (**F**) GRIM-19 inhibited p53 degradation *in vivo*. Before harvesting the cells were treated with MG132 for 4 h, and immunoprecipitation with p53 antibody was performed. Western blotting of the IP products was using ubiquitin antibody. GAPDH antibodies were used to determine the comparable loading.

To further investigate the relationship between GRIM-19 and p53, HeLa cells with either overexpression (pG19) or knockdown (siG19) of GRIM-19 were used, and the levels of GRIM-19 and p53 were evaluated by Western blotting (Figure 2A). Interestingly, when compared to the corresponding control (p/siCon) cells, p53 and its target genes PUMA and p21 increased in HeLa/pG19 cells (Fig. 2A, left panels) and decreased in HeLa/siG19 cells (Figure 2A, right panels). In addition, two other cervical cancer cell lines, SiHa and CaSki, were transfected with either a GRIM-19 expression plasmid or dsRNA targeting GRIM-19 and compared to their respective controls. Results similar to those observed in HeLa were obtained in these cells too (Fig. 2B). Moreover, tests on other HPV-free tumor cell lines (A549 and HO8910) didn't reveal the same results as observed in HeLa (Fig S1). Thus, the levels of GRIM-19 directly correlated with those of p53 in cervical cancer cells.

To investigate whether GRIM-19 increased p53 via transcriptional regulation, p53 mRNA levels were examined by quantitative RT-PCR and luciferase reporter assay were performed in HeLa/pCon and HeLa/pG19 cells. The level of p53 mRNA was unaffected by overexpression of GRIM-19 (Fig. 2C). Additionally, p53 promoter-driven luciferase reporter did not reveal significant changes in p53 promoter activity in HeLa/pCon and HeLa/pG19 cells (Fig. 2D), suggesting that GRIM-19 is not involved in the transcriptional activation of p53.

In cervical tumors, p53 is infrequently mutated [23], and it is rapidly degraded by E6/E6AP [6], [24], we next examined whether increase in p53 levels is due to enhanced half-life of the protein. HeLa/pG19 and HeLa/pCon cells were treated with cycloheximide and p53 protein levels were monitored over time. Indeed, the half-life of p53 protein was significantly (*p*<0.01) prolonged in HeLa/pG19 cells compared to HeLa/pCon cells (Fig. 2E). *In vivo* ubiquitination assay also showed that ubiquitinated p53 in HeLa/pG19 cells was dramatically reduced compared to HeLa/pCon cells (Figure 2F).

Taken together, these results suggested that GRIM-19 restored p53 levels through protein stabilization rather than transcriptional up-regulation in cervical tumors.

### GRIM-19 stabilizes p53 protein by interacting with E6 and E6AP proteins

**Figure 3:**
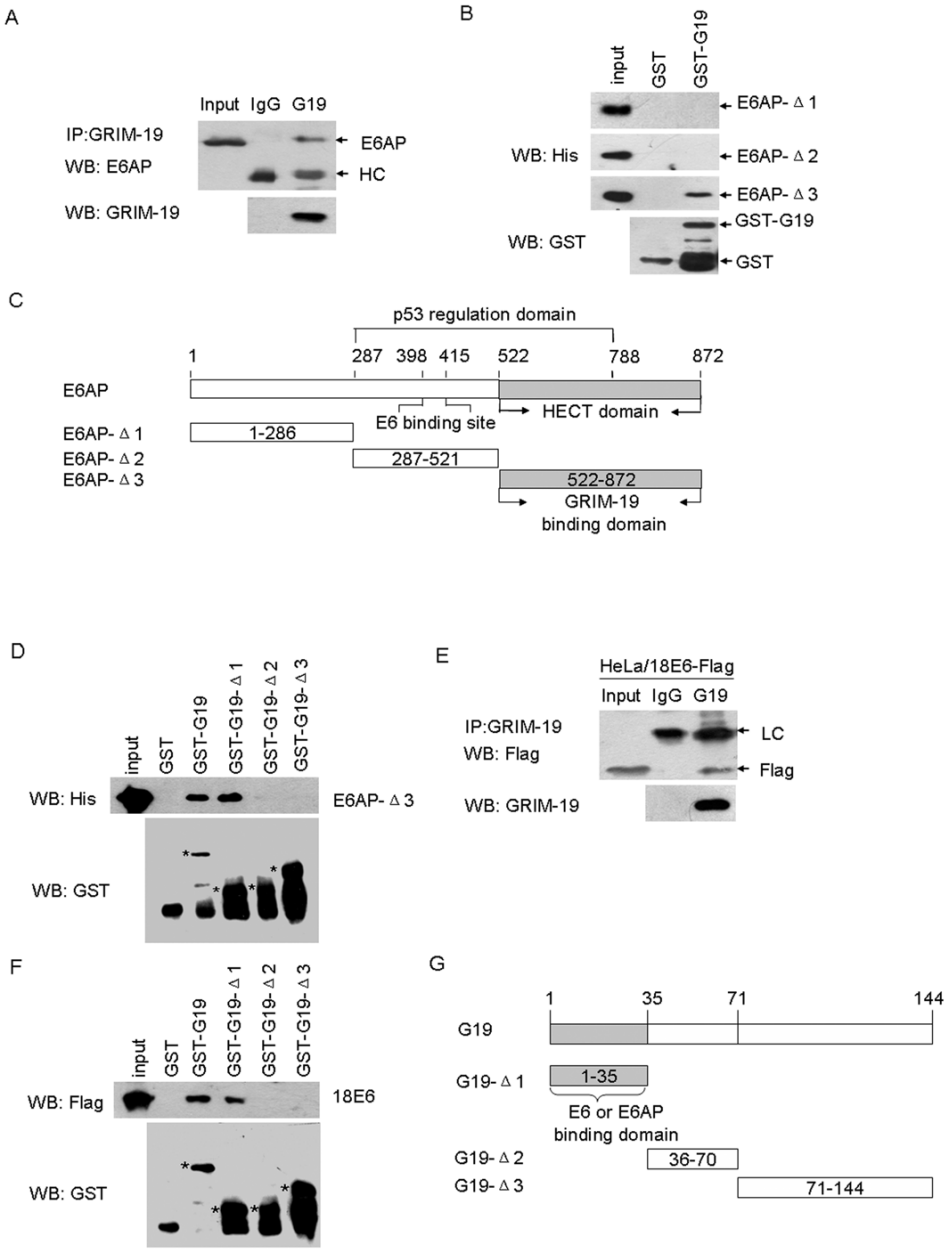
GRIM-19 bound to 18E6 and E6AP protein *in vivo* and *in vitro*. (**A**) Co-immunoprecipitation assays were performed to determine the interaction between GRIM-19 with E6AP *in vivo*. The cell lysates from HeLa cells were immunoprecipitated with normal IgG and anti-GRIM-19 antibodies and Western blotted with anti-E6AP. Input (preIP) lane represents 10% of the extract used in the immunoprecipitation reaction. HC = IgG heavy chain. (**B**) GST pull-down experiments were performed to examine the interaction of His-tagged E6AP deletions with GST-fused GRIM-19 protein *in vitro*. (**C**) Schematic diagram of E6AP indicating various functional domains including the binding sites for GRIM-19. (**D**) The interaction of GST-GRIM-19 deletions with E6AP. * indicates the position of the band with correct molecular size. (**E**) Co-immunoprecipitation assays were performed to determine the interaction between GRIM-19 with 18E6. The cell lysates from HeLa cells transfected with the p18E6-Flag were subjected to IP with the indicated antibodies. Input (preIP) lane represents 10% of the extract used in the immunoprecipitation reaction. LC = IgG light chain. (**F**) The interaction of GST-GRIM-19 deletions with 18E6. * indicates the position of the band with correct molecular size. (**G**) Deletion mapping of the E6 or E6AP binding sites on the GRIM-19.

E6/E6AP-mediated p53 degradation is considered as an important mechanism in initiation and development of cervical carcinomas [23], [25]. Since GRIM-19 binds to 16E6 [26], we hypothesized that GRIM-19 interferes with E6/E6AP complex, thereby protecting p53 from degradation. Using immunoprecipitation assays with cellular lysates from HeLa cells, we found the interaction of GRIM-19 with E6AP *in vivo* (Fig. 3A). We then examined the GRIM-19-E6AP interaction *in vitro* with GST pull-down assays. E6AP encodes three different protein isoforms (I, II and III) that differ in their N terminal tails, all of which have the ability to stimulate E6-mediated ubiquitin-dependent degradation of p53 protein [27]. Because several functional domains of E6AP had been previously identified [28], we generated three recombinant plasmids expressing His-tagged E6AP-III isoform-based deletions: pE6AP-Δ1 (1–286 aa), pE6AP-Δ2 (287–521 aa) and pE6AP-Δ3 (522–872 aa). E6 binding sites were located between aa 287–521 of E6AP-III, while the HECT domain for ubiquitin binding sites was located in the segment from 522–872 aa (Fig. 3C) [29]. We found that only E6AP-Δ3 bound to GST-tagged GRIM-19 by GST pull-down assay (Fig. 3B–C), but not the catalytically inactive E6AP containing a mutation on Cys at position 840 (Fig S2), These results supported our conclusion that GRIM-19 can bind the HECT domain of E6AP. To map the exact interacting region of GRIM-19 with E6AP, we employed GST-tagged GRIM-19 deletions pGST-G19-Δ1, pGST-G19-Δ2 and pGST-G19-Δ3; And found that amino acids 1–35 of GRIM-19 were sufficient for binding E6AP proteins (Fig. 3D).

We have reported the interaction of GRIM-19 with 16E6 before [26], we then explored the association of GRIM-19 and 18E6. Due to the low expression of E6 and poor reactivity of available E6 antibodies that have been reported by a number of publications [30], [31], [32], [33], we failed to obtain a satisfactory 18E6 Western blot from the cell lysates. Therefore, a plasmid p18E6-Flag that expresses a Flag-tagged 18E6 protein was constructed. Through Immunoprecipitation assays in HeLa cells transfected with p18E6-Flag, we found 18E6 co-precipitated with GRIM-19 *in vivo* (Fig. 3E). To map the exact interacting region of GRIM-19 with 18E6, we employed the binding site of GRIM-19 with 18E6 by using GST-tagged GRIM-19 deletions (pGST-G19-Δ1, pGST-G19-Δ2 and pGST-G19-Δ3); And found that amino acids 1–35 of GRIM-19 were sufficient for binding 18E6 proteins *in vitro* (Fig. 3F).

We therefore concluded that GRIM-19 can bind both 18E6 and E6AP *in vivo and in vitro*, which might play a role in accumulation p53 protein.

### GRIM-19 disrupts E6/E6AP complex and augment E6AP ubiquitination and degradation

**Figure 4:**
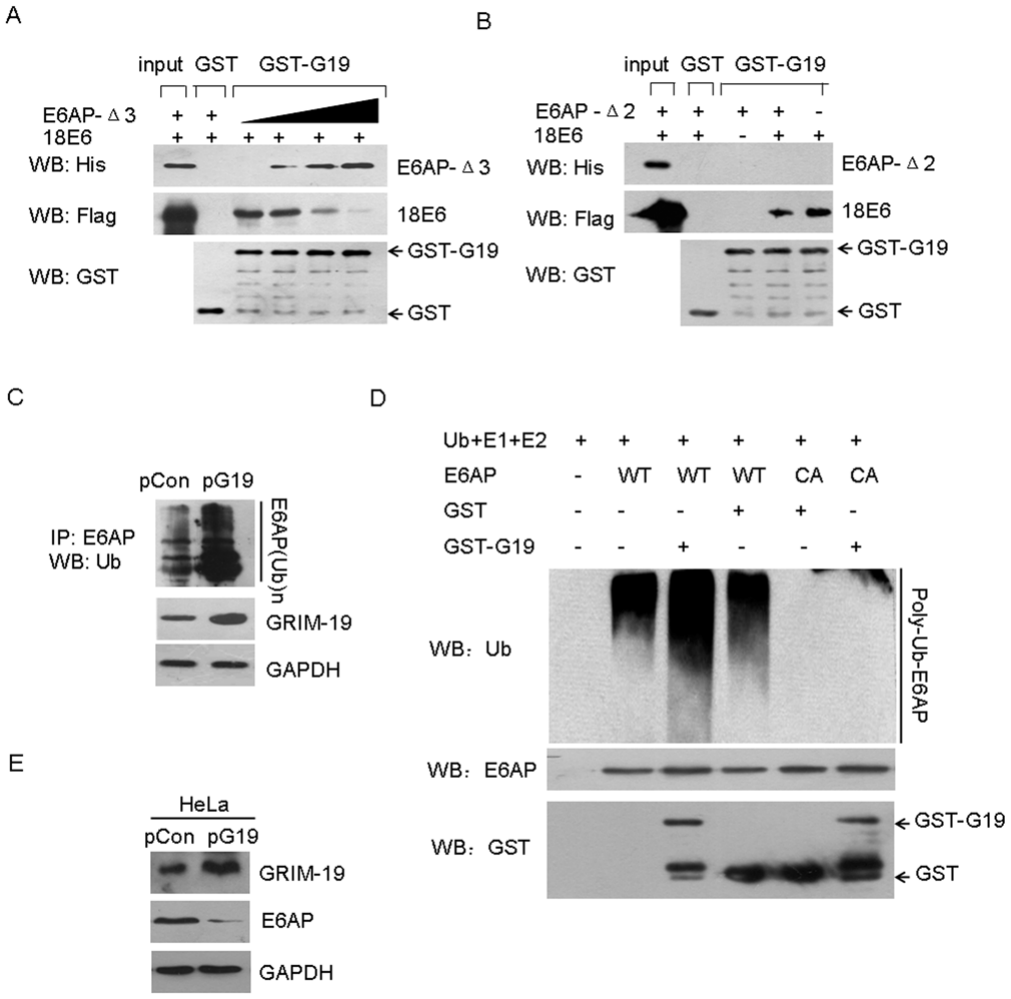
GRIM-19 disrupted E6/E6AP complex and augment E6AP ubiquitination and degradation. (**A**) Competitive assays were performed to analyze the binding of 18E6 to GST-GRIM-19 fusion protein in presence of increasing amounts of E6AP-Δ3 ranging from 0–6 µg. (**B**) Pull-down experiments to determine the binding of 18E6 to GST-GRIM-19 proteins. Where indicated E6AP-Δ2 proteins (10 ug) were added into GST Pull-down reaction. (**C**) GRIM-19 augmented E6AP degradation *in vivo*. Before harvesting the cells were treated with MG132 for 4 h, and immunoprecipitation with E6AP antibody was performed. Western blotting of the IP products was using ubiquitin antibody. GAPDH antibodies were used to determine the comparable loading. (**D**) *In vitro* E6AP ubiquitination assay. Human recombinant ubiquitin, E1, E2 (UbcH5c), batereria-expressed and purified GST and GTS-GRIM-19, E6AP (wild-type or catalytically inactive mutant CA) from wheat germ extract were mixed for *in vitro* E6AP ubiquitination assay and immunoblotted with ubiquitin antibody. (**E**) Whole cell lysates from HeLa cells expressing the indicated expression plasmids were Western blotted with the indicated antibodies.

Given that both 18E6 and E6AP-Δ3 can interact with amino acids 1–35 of N terminus of GRIM-19 (Fig. 3G), we determined whether 18E6 competed with E6AP in binding GRIM-19 by performing competition pull-down assays. In the presence of purified GST-G19 protein and increasing amount of E6AP-Δ3, the binding of 18E6 to GRIM-19 progressively decreased as E6AP-Δ3 increased (Fig. 4A). Although E6AP-Δ2 failed to interact with GST-G19, it is well known that this region harbors E6-binding sites [28]. Therefore, we tested whether GST-G19 and E6AP-Δ2 compete in binding with 18E6. In the presence of E6AP-Δ2, GST-G19-bound 18E6 decreased compared to 18E6 presented alone (Fig. 4B). Furthermore, we did not observe an association of E6AP-Δ2 with GST-G19 in the presence of 18E6, suggesting that the proteins cannot form a heterotrimeric complex of 18E6/E6AP-Δ2/GST-G19.

It has been well established that E6AP can target itself for ubiquitination, which represents a mechanism to control its own half-life [7], [34]. We then explore if the expression of GRIM-19 can influence the degradation of E6AP, in the presence of overexpressed GRIM-19, ubiquitinated E6AP significantly increased compared to HeLa/pCon cells (Fig. 4C). In vitro ubiquitination assay showed that GRIM-19 increased the autoubiquitination of wild-type E6AP, but not its dominant-negative CA mutant by using purifed E1 and E2 (UbcH5c), bacteria-expressed GRIM-19, and wild-type E6AP and CA mutant E6AP translated in wheat germ extract system (Fig. 4D). Indeed, we found E6AP protein was decreased in cells with overexpression GRIM-19 (Fig. 4E, S1).

In summary, we conclude that GRIM-19 prevents p53 degradation by impeding E6/E6AP complex formation and promotes ubiquitination and degradation of E6AP.

### GRIM-19 delays G0/G1 transition, inhibits cell proliferation and induces apoptosis, and promoted p53 accumulation in vivo

**Figure 5:**
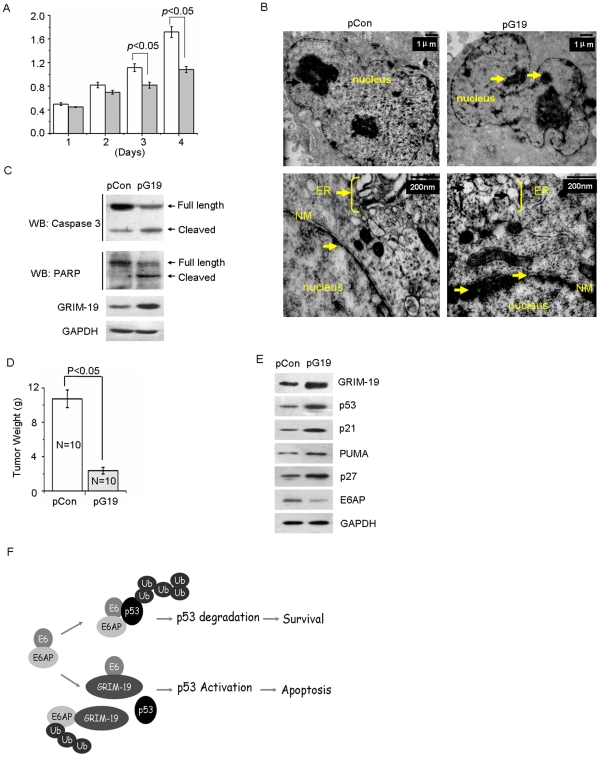
GRIM-19 inhibited cell proliferation and induced apoptosis, and promoted the accumulation of p53 in tumors. (**A**) An MTT assay was performed in the indicated cells. MTT assay was performed as in Materials and Methods. Each data point represents the mean ± SE of 8 samples. (**B**) The morphological characteristics of HeLa/pCon and HeLa/pG19 cells were examined by transmission electron microscopy. Chromatin condensation, expansion and widened nuclear membrane gaps, vague nuclear membrane structure, fractures of nuclear membrane and endoplasmic reticulum expansion were indicated with arrows. ER, endoplasmic reticulum; NM, nuclear membrane. (**C**) The full length and cleaved form of caspase-3 and PARP in HeLa/pCon and HeLa/pG19 cells were determined by Western blot analyses. (**D**) HeLa/Con and HeLa/G19 cells were transplanted into 6-week-old female athymic nude mice (10 mice for each cell line) and grown for 6 weeks. Tumors were harvested, and weights were measured. The data present the mean of 10 tumors in each group. (**E**) The expression of GRIM-19, p53, p21, PUMA, p27 and E6AP in the tumors derived from mice as determined by Western blot analyses, and the representative results are presented. (**F**) A model for the collaboration between GRIM-19 and p53. When GRIM-19 is present in high levels, it interacts with the E6/E6AP complex, promotes their ubiquitination, thus, preventing p53 degradation. The loss of GRIM-19 allows the attack of E6/E6AP complex on p53 and its degradation through the proteasome.

Given that inductions of cell cycle arrest and cell growth suppression are the major functions of p53, we next evaluated the effect of GRIM-19-dependent p53 stabilization on cell cycle. Cell cycle distribution analyses revealed a significantly delayed G0/G1 transition in HeLa/pG19 cells when compared to HeLa/pCon cells (Table 1). Additionally, as indicated by MTT assay, the cell proliferation of HeLa/pG19 cells was significantly (p<0.05) suppressed on day 3 and day 4 (Fig. 5A) compared to HeLa/pCon cells.

**Table 1:** GRIM-19 delayed G0/G1 transition.

Cells	Phase/stage	Time (h)					
		0	4	6	8	10	14
HeLa/pCon	G0/G1	55.54	12.27	10.55	15.50	12.05	72.15
	S	38.53	70.90	51.22	46.30	48.82	7.80
	G2/M	5.93	16.83	38.23	38.20	39.13	20.05
HeLa/pG19	G0/G1	60.20	25.71	18.25	17.66	17.95	66.12
	S	32.80	58.59	49.37	48.01	48.75	7.86
	G2/M	7.00	15.70	32.38	34.33	33.30	26.02

Because promoting cell apoptosis is another key function of p53, we performed cell apoptosis assays including transmission electron microscopy (TEM) and Western blotting with caspase 3 and PARP antibodies. A range of early apoptosis characteristics were indicated in HeLa/pG19 cells, including nuclear chromatin condensation, widened nuclear membrane gap, vague nuclear membrane structure, fractures of nuclear membrane and endoplasmic reticulum expansion; however, these phenomena were not observed in HeLa/pCon cells (Fig. 5B arrows). In addition, the cleavage of caspase 3 and PARP was increased in HeLa/pG19 cells compared to HeLa/pCon cells (Fig. 5C).

We also found the tumor weights in the mice transplanted with HeLa/pG19 cells was significantly reduced compared to those of control groups (*p*<0.05) (Fig. 5D). GRIM-19 and p53, together with p21, PUMA and p27 proteins were significantly increased, but E6AP was decreased in the tumors derived from HeLa/pG19 cells compared to tumors from HeLa/pCon cells (Fig. 5E).

Finally, based on our observations, a model for GRIM-19 induced cell apoptosis by disrupting the E6/E6AP complex and stabilizing p53 was speculated (Fig. 5F). This model suggests that the presence of GRIM-19 promotes auto-ubiquitination and degradation of E6AP by interacting with these proteins and contributes to p53 stabilization, growth arrest and apoptosis.

## Discussion

High-risk human papillomaviruses (HR-HPV), such as HPV18 and HPV16, are associated with 99.7% of cervical cancers [35], which are the most common gynecologic tumors in developing countries [36]. The viral oncoproteins E6 and E7 are expressed in HPV-positive cervical carcinomas [6], while the viral E2 protein represses transcription of the E6/E7 oncogenes and activates viral DNA replication together with the viral E1 helicase [37], [38], [39]. The E6/E6AP mediated degradation of p53 is considered a most important mechanism in the initiation and development of cervical cancers [6], [11], [12], [25], [40], [41]. Recent studies suggested that interference of the E6/E6AP complex may kill cervical tumors by increasing the level of p53 protein [11], [12], [40]. In our study, we present a new approach to prevent p53 degradation by restoration of GRIM-19 that disrupts the E6/E6AP complex.

It has been reported that GRIM-19 can suppress transcriptional activity of STAT3 through protein-protein interaction, and inhibit cancer growth [16], [17]. STAT3 has been shown to inhibit p53 expression via a transcriptional repression in the src oncogene-induced signaling pathways in certain rodent cell lines [42]. Some studies in cancer cell lines showed a correlation between high constitutive STAT3 activity and p53 gene mutations, although the cause and effect relationships were not established [43]. In certain head and neck squamous cell carcinomas, p53 status has been shown down regulate NF-κB and STAT3 induced gene expression [44]. We have shown in our previous publication that GRIM-19 loss correlates with a high STAT3 activity in primary cervical cancers [20]. Although such observations suggest a possibility that loss of GRIM-19 promotes high STAT3 activity which could ultimately transcriptionally down regulate p53 expression, our studies did not reveal (fig. 2C &D) any changes in the expression of luciferase reporter driven by human p53 promoter and of endogenous p53 mRNA. Therefore, STAT3 deregulation may not have a direct consequence on p53 expression in our model. Thus, two GRIM-19 independent pathways: 1) an activation of STAT3 and 2) a down regulation of p53 concurrently occur in HPV transformed cervical carcinomas for promoting tumorigenesis following the loss of GRIM-19 expression. More importantly, Interference with STAT3 expression using RNA*i* or its function using dominant negative approaches (Fig S3) did not significantly alter the GRIM-19 effects on p53 stabilization. Therefore, p53 protein stabilization by GRIM-19 appears to occur independently of STAT3.

In this paper, we also tested whether GRIM-19 inhibits p53 degradation in the absence of E6. Two HPV negative cells HO8910 and A549 that both containing wild-type p53 [45] were examined for their p53 expression after overexpression of GRIM-19. Although GRIM-19 was able to augment the degradation of E6AP in these two cells, the protein levels of p53 remained unchanged (Fig S1), suggesting that GRIM-19 is able to prevent E6-dependent p53 degradation.

Recent reports have demonstrated that GRIM-19 can interact with some other important physiological proteins such as HtrA2 [46]; Moreover, viral proteins such as U95 and vIRF1 can also bind to GRIM-19 [21], [26], [47]. We have previously shown an association between 16E6 and GRIM-19 [26]; here, we also found the interaction of GRIM-19 with 18E6 and E6AP *in vivo* and *in vitro*, and the induction of the autoubiquitination degradation of E6AP by GRIM-19. In HR-HPV infection cervical cancer cells, GRIM-19 was able to induce accumulation of the p53 protein and to increase p53 target genes such as p21 and PUMA. Additionally, significant changes on the cell cycle profile, cell proliferation, and the characteristic morphological signs of apoptosis were observed in HeLa cells overexpression of GRIM-19. Thus, GRIM-19 and p53 can synergistically suppress cervical cancer cell growth.

E6AP is a critical regulator of p53 degradation in human cervical cancers in an E6 dependent manner. Apart fromp53, E6/E6AP complex has been reported to interfere with several cellular functions [48] including transcriptional activators such as IRF3, co-activators such asp300, apoptosis inducers such as Bak, GADD34, procaspase-8 and its adaptor FADD, protein kinases such as tyk2, cell adhesion associated molecules such as Paxillin, and NFX1-91, a molecules that attenuates telomerase activity. In most of these cases the E6/E6AP complex targets these proteins to degradation to allow oncogenic transformation [48]. E6 interaction with E6AP has been reported to be important for skin carcinogenesis in transgenic mouse models [49], [50]. E6AP also targets proteins in an E6-independent manner. In fact, several substrates such as members of the Src family of protein kinases [51], polycomb protein Ring1B [52] and the promyelocytic leukemia (PML) protein [53] have been reported. Naturally occurring sporadic E6AP mutations are associated with Angelman's syndrome, a severe form of mental retardation, wherein accumulation of undegraded protein aggregates has been reported [52], [54], [55], [56]. Thus, E6AP protein in association with E6 and in some situations on its own plays a central role in protein degradation controlling several human pathologies.

Because E6AP also acts as a dual function coactivator for steroid hormone receptors (SHRs) including progesterone receptor, estrogen receptor, androgen receptor, glucocorticoid receptor, retinoic acid receptor-α and thyroid hormone receptor [29], [57], and the amino acids from 170–680 are the activation domain of E6AP [29], the interaction of GRIM-19 with E6AP (522–872 aa) might predict that GRIM-19 probably regulates SHR-dependent gene transcription.

In summary, our studies for the first time show a novel mechanism by which GRIM-19 blocks E6/E6AP complex; and the collaboration between two distinct tumor suppressor proteins in regulating cell growth.

## Materials and Methods

### Ethics Statement

All cervical tissues were obtained from patients who underwent hysterectomy between January 2008 and November 2009 at Anhui Provincial Hospital affiliated to Anhui Medical University, Hefei, China. The study was reviewed and approved by the ethics review board of Anhui Provincial Hospital. Written consent was obtained from each patient.

All animal experimental procedures carried out in this study have been approved by the Laboratory Animal of the Ethics Committee of Anhui Provincial Hospital Affiliated to Anhui Medical University under permit number 201000179, and were in compliance with the guidelines for animal care set forth by this Committee.

### Tumors

A portion of freshly-excised tissues were paraffin-embedded, cut into 5- to 7-µm-thick sections for pathologic diagnosis, and the rest of the tissue was frozen at −80°C for further use to extract proteins and RNA. Clinical stages were determined by a certified gynecologic pathologist according to a modified International Federation of Gynecology Obstetrics (FIGO) staging system for cervical cancer in 2000. The 60 non-metastatic squamous epithelial carcinomas examined in these studies were HPV16 or HPV18 positive and belonged to type Ia (13 patients), Ib (9 patients) and IIa (38 patients). Additionally, 45 normal cervical tissues from patients who underwent hysterectomy for reasons other than neoplasia of either the cervix or endometrium were collected and used as normal controls in this study.

### Cell culture and transfection

Human cervical cancer cell lines HeLa, SiHa and CaSki and the human lung adenocarcinoma cell line A549 from American Type Culture Collection (ATCC) were grown DMEM with 10% fetal bovine serum. The human ovarian cancer cell line HO8910 was purchased from cell bank of the Chinese Academy of Sciences [58], [59] and grown in complete RPMI-1640. Lipofectamine 2000 (Invitrogen) was used for transfection. The stably transfected cell lines HeLa/pCon and HeLa/pG19 expressing control vector and human GRIM-19, respectively, were described previously [20].

### Tumor xenografts

Animals were reared under standard laboratory conditions. Two groups (10 in each group) of 6-week-old female athymic nude mice (Beijing experimental animal center) were implanted subcutaneously on the right flank of mice with either HeLa/pG19 or HeLa/pCon cells (1×10^7^) in 0.1 ml PBS containing 50% matrigel. All mice were housed in a pathogen-free environment. At the end of the experiment (6 weeks after implantation), mice were euthanized, tumors were collected and weighed. A portion of each tumor was processed for immunohistochemical and biochemical analyses, and the rest was frozen at −80°C until use.

### Plasmids

The plasmids LZRSpBMN-linker-IRES-EGFP-STAT3C (STAT3C) expressing a constitutively active mutant STAT3 and LZRS pBMN-linker-IRES-EGFP-STAT3DN (STAT3DN) expressing a dominant negative mutant STAT3 were gifts from Dr. Hodge DR as described previously [60]. The empty vector pIRES-Puro2-Myc (pCon) and pIRES-Puro2-GRIM-19-Myc (pG19) expressing a Myc-tagged GRIM-19 were described previously [20]. The luciferase reporter pGL3-P53-Luc containing a 676-bp human p53 promoter sequence from −29 bp to −704 bp of the ATG start site was PCR amplified with human genome DNA (Invitrogen) by using a sense primer with a SacI restriction enzyme site (underlined) 5′-GAGCTCCAACAATGAATAAGATACTAG-3′ and an antisense primer with a BglII restriction enzyme site (underlined) 5′-AGATCTCAATCCAGG GAAGCGTGTCAC-3′ and was subsequently ligated into SacI/Bgl II-double digested pGL3-basic vector (Promega).

To construct a plasmid expressing a wild-type HPV18 E6 protein tagged with Flag, the coding sequences were amplified with genome DNA from HeLa cell as template (GeneBank Accession: X05015) by a primer pair: a sense primer with an HindIII restriction enzyme site (underlined): 5′-CCCAAGCTTG CGCGCTTTGAGGATCCAA-3′; and an antisense primer with a KpnI restriction enzyme site (underlined): 5′-CGGGGTACCTTATA CTTGTGTTTCTCTGCGTCG-3′. The PCR products were inserted into a HindIII/KpnI -digested p3×Flag-Myc-CMV™-24 vector (Sigma). The resulted clone was designed as p18E6-Flag.

To construct the bacterial expression vector for Flag-tagged HPV18 E6 protein, p18E6-Flag was used as the template and PCR amplified with the primer pair: a sense primer with an NdeI restriction enzyme site (underlined): 5′-GGGAATTCCATATGGACTACAAAGACCATGA CGG-3′; and an antisense primer with a Xhol restriction enzyme site (underlined): 5′-CCGCTCGAGTTATACTTGTGTTTCTC TGCGTCG-3′. The PCR products were inserted into an NdeI/Xhol-digested pET-22b vector (Novagen) and the resulted plasmid was designed as pET-18E6-Flag.

To construct the bacterial recombinant plasmid expressing His-tagged wild-type E6AP protein, the coding sequence of E6AP was PCR amplified using pCMV6-XL5-E6AP-III (Origene, SC120518, USA,) as a template with the following primer pair: a sense primer with an NdeI restriction enzyme site (underlined): 5′-GGGAATTCCATATGGCCACAGCTTGTAAAAGA TCAGG -3′; and an antisense primer with a HindIII restriction enzyme site (underlined): 5′-CCCAAGCTTCA GCATGCCAAATCCTTTGG -3′. The PCR products were inserted into an NdeI/HindIII -digested pET-22b vector (Novagen), and the resulted plasmid was designed as pET-E6AP-his.

By using pET-E6AP-his as the template, the E6AP deletions were PCR-amplified to construct the truncated His-tagged E6AP-expressing vectors (Figure 3C). The primers for pE6AP-Δ1 (1–286 aa): a sense primer with an NdeI restriction enzyme site (underlined): 5′-GGGAATTC CATATGGCCACAGC TTGTAAAAGA TCAGG -3′; and an antisense primer with a HindIII restriction enzyme site (underlined): 5′-CCCAAGCTTGATAATGAACAAATTCAGA -3′. The primers for pE6AP-Δ2 (287–521 aa) were: a sense primer with an NdeI restriction enzyme site (underlined): 5′-GGGAATTC CATATGGTAATGGAGAATAGAAATCTCC-3′; and an antisense primer with a HindIII restriction enzyme site (underlined): 5′-CCCAAGCTTTCTCAA ATATGGATTCAACT-3′. The primers for pE6AP-Δ3 (522–872 aa) were: a sense primer with an NdeI restriction enzyme site (underlined): 5′-GGGAATTCCATATGCTCAAAG TTAGACGTGACCA-3′; and an antisense primer with a HindIII restriction enzyme site (underlined): 5′-CCCAAGCTTCAGCATGCC AAATC CTTT-3′. The PCR products were inserted into an NdeI/HindIII -digested pET-22b vector.

To construct the bacterial recombinant plasmid expressing GST-tagged GRIM-19, the coding sequences were PCR amplified using pIRES-Puro2-GRIM-19-Myc as a template with the following primer pair: a sense primer with a BamHI restriction enzyme site (underlined): 5′-CGCGGATCCGCGGCGTC AAAGGTGAAG -3′; and an antisense primer with an Xhol restriction enzyme site (underlined): 5′-CCGCTCGAGCTACGTGTACCACATGAA GCC -3′. The PCR products were inserted into a BamHI/Xhol -digested pGEX-4T-3 vector (GE Healthcare, Piscataway, NJ, USA) to construct pGST-G19.

By using pGST-G19 as a template, GRIM-19 deletions were PCR-amplified to construct the truncated GRIM-19-expressing vector. The primers for pGST-G19-Δ1 (1–35 aa): a sense primer with a BamHI restriction enzyme site (underlined): 5′- CGCGGATCCGCGGC GTCAAAGGTGAAG -3′, and an antisense primer with an XhoI restriction enzyme site (underlined): 5′- CCGCTCGAGCTACATGCTGTAGCCCGA -3′. The primers for pGST-G19-Δ2 (36–70 aa) were: a sense primer with a BamHI restriction enzyme site (underlined): 5′- CGCGGATCCCTGGCCATAGGGATTGGAA -3′, and an antisense primer with an XhoI restriction enzyme site (underlined): 5′- CCGCTCGAGCTACGCGATGCGAGCCT -3′. The primers for pGST-G19-Δ3 (71–144 aa) were: a sense primer with a BamHI restriction enzyme site (underlined): 5′- CGC GGATCCCTGTTGCCACTGTTACAGGC AA-3′; and an antisense primer with an XhoI restriction enzyme site (underlined): 5′- CCGCTCGAGCTACGTGTACC ACATGAAGCC -3′. The PCR products were inserted into a BamHI/XhoI-digested pGEX-4T-3 vector. All of the plasmids were confirmed by DNA sequencing.

To construct a plasmid encoding a catalytically inactive mutant E6AP-III, plasmids of pGEX-2T-E6AP-I C833A (provided by Dr. Peter Howley, Harvard Medical School, Boston, MA) and pCMV6-XL5-E6AP-III were used as templates, and the sequences for E6AP-III mutant were amplified with primer pairs: a sense primer with a BamHI restriction enzyme site (underlined): 5′-CGCGGATCCGCCACAGCTTGTAAAAGA TCAGG -3′; and an antisense primer with an Xhol restriction enzyme site (underlined): 5′-CCGCTCGAGTCTCAAATATGGATTCAACT -3′; another sense primer with a BamHI restriction enzyme site (underlined): 5′-CGCGGATCC CTCAAAGTTAGACGTGACCA -3′; and another antisense primer with an XhoI restriction enzyme site (underlined): 5′-CCGCTCGAG CAGCATGCC AAATC CTTT -3′. The PCR products were inserted into a BamHI/XhoI -digested pGEX-4T-3 vector (GE Healthcare), and the recombinant plasmid was named as pGST-CA-E6AP.

To construct a plasmid encoding catalytically inactive mutant E6AP-III with T7 promoter for *in vitro* translation, the mutant E6AP-III sequence was PCR amplified using pGST-C840A-E6AP as a template with the following primer pair: a sense primer with an NdeI restriction enzyme site (underlined): 5′-GGGAATTCCATATGGCCACAGCTTGTAAAAGATCAGG -3′; and an antisense primer with a HindIII restriction enzyme site (underlined): 5′-CCCAAGCTTCAGCA TGCCAAATCC TTTGG -3′. The PCR products were inserted into an NdeI/HindIII-digested pET-22b vector (Novagen), and the recombinant plasmid was named as pET- CA -E6AP.

To construct the bacterial recombinant plasmid expressing Flag-tagged GRIM-19, the coding sequences were PCR amplified using pIRES-Puro2-GRIM-19-Myc as a template with the following primer pair: a sense primer with a HindIII restriction enzyme site (underlined): 5′-CCCAAGCTTCAAG AACCAAGGCGAGTCA-3′; and an antisense primer with a BamHI restriction enzyme site (underlined): 5′-CGCGGATCCTACGTGTACCACATGAAGCCG-3′. The PCR products were inserted into a HindIII/BamHI-digested p3×Flag-Myc-CMV™-24 vector to construct pGRIM-19-Flag. Then pET-GRIM-19-Flag was amplified using pGRIM-19-Flag as a template with the following primer pair: a sense primer with a NdeI restriction enzyme site (underlined): 5′-GGGAATTCCATATGGACTA CAAAGACCATGACGG-3′; and an antisense primer with a HindIII restriction enzyme site (underlined): 5′-CCCAAGCTTCTACGTGTACCACATGAAGCCG-3′. The PCR products were cloned into a NdeI/HindIII-digested pET-22b vector to obtain pET-GRIM-19-Flag.

### siRNA

The STAT3 siRNA sequence was: 5′- CCAACGACCUGCAGCAAUAUU-3′. The GRIM-19 siRNA sequence was: 5′- GCUUCAUGUGGUACACGUATT-3′. In addition, an siRNA with a random sequence (5′- UUCUCCGAACGUGUCACGUTT-3′) was used as the scrambled control. All of the plasmids were confirmed by DNA sequencing.

### Reverse transcription and quantitative PCR

One microgram total RNA extracted from tissues or cells was converted to cDNA using Superscript III reverse transcriptase (Invitrogen). Quantitative PCR employing SYBR chemistry (Sigma) was conducted using the primer sets indicated in Table S1.

### Immunoblotting

Proteins were transferred to Polyvinylidene fluoride (PVDF) membranes and probed with the indicated primary antibodies. GAPDH, p21, STAT3, pY-STAT3, p27 antibodies were purchased from Cell Signaling. GRIM-19, Ubiquitin, PARP, Caspase-3 and p53 antibodies were from Santa Cruz. PUMA, Flag, His, E6AP antibodies were from Sigma. GST antibody was from GE Healthcare. Membranes were then incubated with a 1∶5000 dilution of a peroxidase-conjugated corresponding secondary antibody (Sigma). Blots were developed using an Enhanced Chemiluminescence kit (Pierce).

### Luciferase assays

Briefly, pGL3-P53-Luc and Renilla luciferase (internal control) plasmids were co-transfected into cells. Luciferase activity was determined using a dual-luciferase reporter assay kit (Promega). The p53-Luc plasmid contains a 676 bp fragment of the human p53 promoter (from +29 to −704 bp). The p53- luciferase activity was normalized to that of Renilla luciferase (internal control). The data represent at least three independent experiments. Triplicate samples were analyzed in each experiment,

### Determination of p53 half-life

After overnight growth in complete RPMI-1640, cells (2×10^6^) were treated with 100 mg/ml CHX (Sigma) for the indicated times, washed with ice-cold 1×PBS and lysed in RIPA buffer. Equal quantity of protein from each sample was subjected to immunoblotting band. The intensities of p53 protein and GAPDH were quantified and the ratio was plotted as a percentage of remaining p53. Triplicate samples were analyzed in each case; and the average of three experiments was used to calculate the half-life of p53.

### Synchronization and cell cycle analyses

The cells grown in serum-rich medium were synchronized by incubating with 2.5 mM of thymidine (Sigma) for 18 h. Medium was changed and they were grown for additional 8 h, after which they were once again synchronized by incubating with 2.5 mM thymidine for a further 18 h. At the end of synchronization, cells were washed and cultured in a fresh serum-rich medium for the indicated periods. They were then stained with Propidium Iodide (BD Biosciences) and analyzed by flow cytometry.

### MTT assay

Cells (2×10^3^/well) were seeded into a 96-well plate and incubated at 37°C and growth was measured by adding 20 µl of MTT (5 mg/ml) into the culture medium. MTT reduction was quantified using a plate reader. All assays were performed in triplicate, and the data are presented as the mean ± SD.

### Transmission electron microscopy

For the morphologic studies, 1×10^7^ cells were fixed with 2.5% glutaraldehyde in 0.1 mol/L phosphate-buffered saline (PBS), post-fixed with 1% osmium tetroxide, and embedded in Epon according to routine techniques. Thin sections were mounted on nickel grids and examined by transmission electron microscopy after staining with uranylacetate and lead citrate.

### Coimmunoprecipitation

Clarified cellular lysates were incubated with the indicated primary antibodies or normal mouse IgG (Santa Cruz) for 2 h at 4°C followed by the addition of Protein G-agarose beads for 2 h at 4°C. The bound complexes were then washed thrice with RIPA buffer and separated by SDS-PAGE. Immunoblotting was performed as described above.

### In vitro translation

*In vitro* translation was performed using the T_N_T Quick Coupled Transcription/Translation System (Promega) following the manufacturer's protocol. Briefly, 1 µg of plasmids were incubated with 25 µl of wheat germ extracts, 2 µl reaction buffer, 1 µl RNA polymerase (T7), 0.5 µl 1 mM amino acid mixture minus leucine, 0.5 µl 1 mM amino acid mixture minus methionine, 1 µl ribonuclease inhibitor, 1 µl transcend™ tRNA at 30°C for 90 min. Nuclease-free water was added to a final volume of 50 µl.

### GST pull-down

The recombinant proteins were expressed in *E.coli* BL21 strain and were affinity purified with glutathione-Sepharose 4B resin (GE Healthcare). For pull-down assays, 10 µg of GST or GST fusion proteins were immobilized on glutathione-agarose for 2 h at 4°C and were washed with cold PBS containing 1% TritonX-100. The GST protein immobilized on glutathione-agarose was then incubated with the cell lysates from *E. coli* BL21 strains expressing Flag-tagged or His-tagged protein at 4°C overnight and subsequently washed with cold 1×PBS containing 1% TritonX-100. The bound proteins were eluted and subjected to Western blotting with the indicated antibodies.

### In vivo ubiquitination assays

Cells were treated with the proteasome inhibitor MG-132 (20 µM; Calbiochem) for the indicated times, Cell extract (1 mg) from each sample was immunoprecipitated with anti-ubiquitin or the indicated specific antibody followed by Protein G agarose. After washing with RIPA buffer, the immunocomplexes were resolved by SDS-PAGE, Western blotted and probed with a monoclonal anti-ubiquitin antibody.

### In vitro ubiquitination assays

GST, GST-GRIM-19 were expressed in E.coli BL21 and purified. Wild-type E6AP and dominant mutant C840A E6AP were translated in wheat germ extracts with pCMV6-XL5-E6AP-III and pET- C840A-E6AP respectively. To assess the *in vitro* autoubiquitination of E6AP, the assays were performed in 30 µl ubiquitination assay buffer (50 mM Tris-HCl ph8.0, 50 mM NaCl, 1 mM dithiothreitol, 5 mM MgCl_2_, 3 mM ATP), with 0.25 µg of E1, 0.25 µg of UbcH5c (E2), 10 µg of ubiquitin (all from Boston Biochem, Cambridge, Massachusetts, USA), 10 µl translated E6AP (wild-type or C840A mutant) in wheat germ extracts and 1.5 µg of GST or GST-GRIM-19. Samples were incubated at 30°C for 2 h and reactions were terminated with 25 mM EDTA before Western blotting with anti-Ubiquitin antibody.

### Statistical analysis

The SPSS13.0 software package (SPSS, Inc, Chicago, IL) was used for all statistical analyses. Statistical significance was evaluated using the Student's *t*-test for paired comparison with a *p* value of <0.05 considered statistically significant.

## Supporting Information

Figure S1 **GRIM-19 did not induce p53 protein accumulation in non- E6-harboring cells.** Cell lysates from HO8910 or A549 cells with overexpression GRIM-19 together with their corresponding controls were used for western blot analysis.
(TIF)

Figure S2 **GRIM-19 did not bind to mutant CA E6AP** ***in vitro*** **.** GST pull-down experiments were performed to examine the interaction of GST-fused mutant CA E6AP with Flag-tagged GRIM-19 protein *in vitro*.
(TIF)

Figure S3 **GRIM-19-induced p53 protein accumulation is independent of STAT3.** (**A**) HeLa and SiHa cells were transfected with either control siRNA or STAT3 siRNA. Forty-eight hours later, cell lysates were prepared and subjected to western blot analysis with the indicated antibodies. (**B**) HeLa and SiHa cells were transfected with either STAT3C,STAT3DN expression vectors or the control vector. Forty-eight hours later, cell lysates were prepared and subjected to western blot analysis with the indicated antibodies.
(TIF)

Table S1 List of oligonucleotides used in this study.
(DOC)
