# Supplementary figures and images for: GRIM-19 Disrupts E6/E6AP Complex to Rescue p53 and Induce Apoptosis in Cervical Cancers
Source: PLoS One. 2011 Jul 12;6(7):e22065. doi: 10.1371/journal.pone.0022065 (PMC3134474; doi:10.1371/journal.pone.0022065)

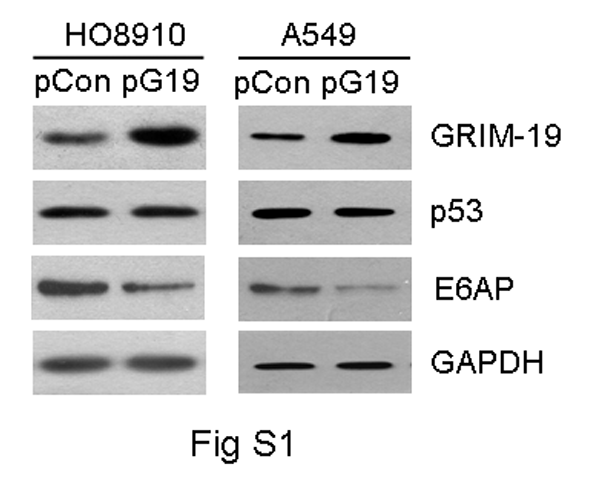

Supplement: Figure S1 — GRIM-19 did not induce p53 protein accumulation in non- E6-harboring cells. Cell lysates from HO8910 or A549 cells with overexpression GRIM-19 together with their corresponding controls were used for western blot analysis. (TIF) [file pone.0022065.s001.tif]

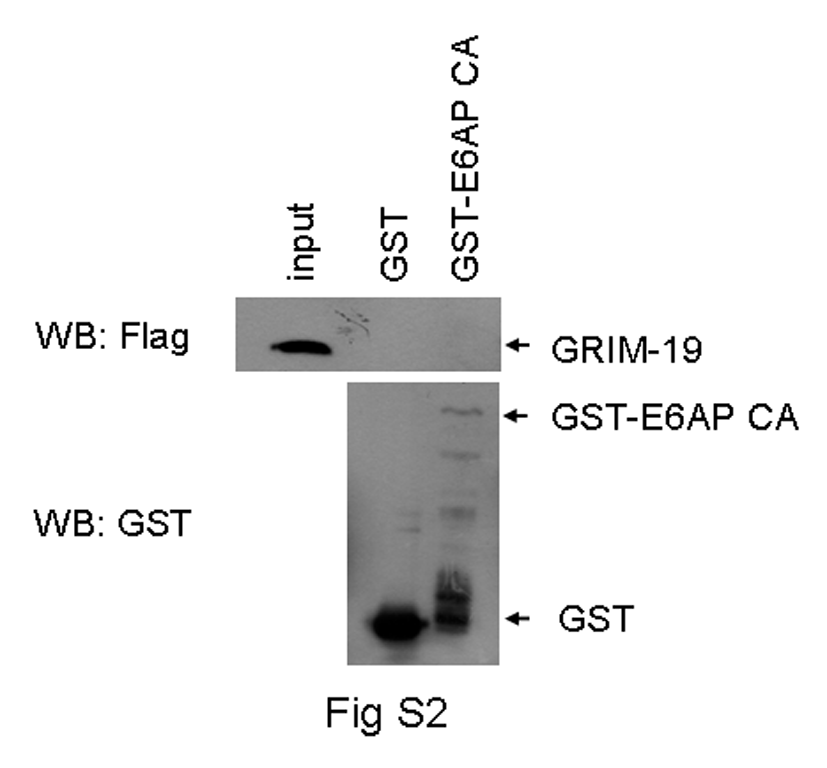

Supplement: Figure S2 — GRIM-19 did not bind to mutant CA E6AP in vitro . GST pull-down experiments were performed to examine the interaction of GST-fused mutant CA E6AP with Flag-tagged GRIM-19 protein in vitro. (TIF) [file pone.0022065.s002.tif]

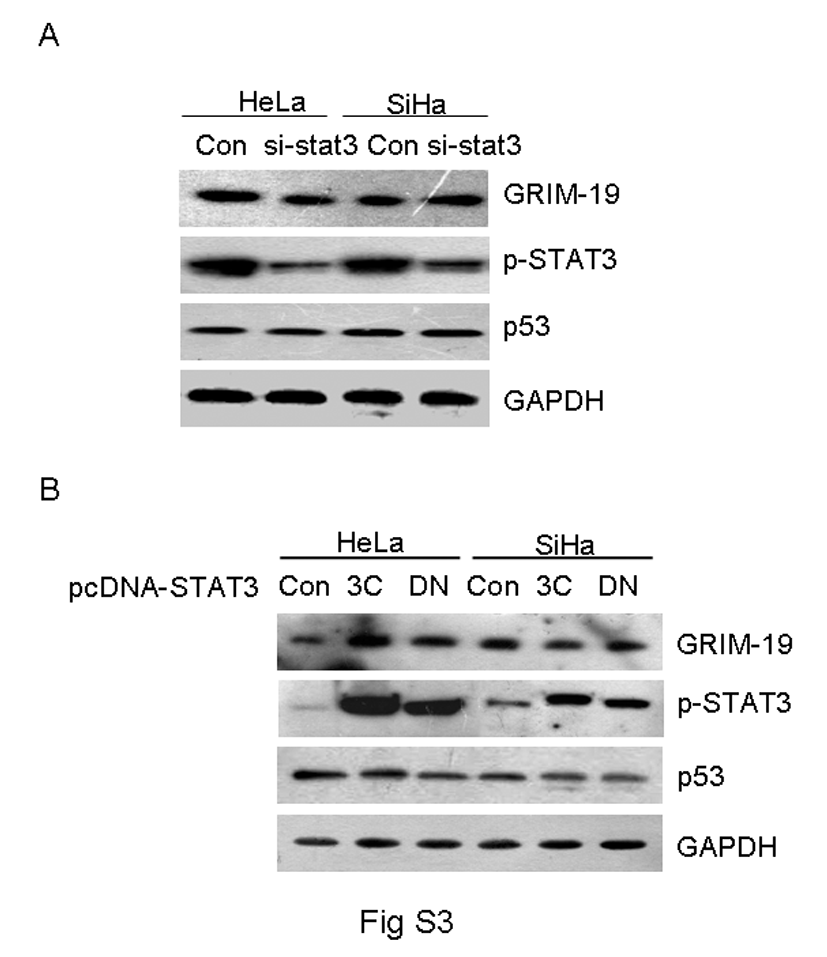

Supplement: Figure S3 — GRIM-19-induced p53 protein accumulation is independent of STAT3. (A) HeLa and SiHa cells were transfected with either control siRNA or STAT3 siRNA. Forty-eight hours later, cell lysates were prepared and subjected to western blot analysis with the indicated antibodies. (B) HeLa and SiHa cells were transfected with either STAT3C,STAT3DN expression vectors or the control vector. Forty-eight hours later, cell lysates were prepared and subjected to western blot analysis with the indicated antibodies. (TIF) [file pone.0022065.s003.tif]
