# Supplementary material for: GRIM-19 Disrupts E6/E6AP Complex to Rescue p53 and Induce Apoptosis in Cervical Cancers
Source: PLoS One. 2011 Jul 12;6(7):e22065. doi: 10.1371/journal.pone.0022065 (PMC3134474; doi:10.1371/journal.pone.0022065)
Supplement: Table S1 — List of oligonucleotides used in this study. (DOC) [file pone.0022065.s004.doc]

Table S1. List of oligonucleotides used in this study

| Transcript | Primer sequence | Product size (bp) |
| --- | --- | --- |
| GRIM-19  GAPDH  p53  E6AP  HPV18E6 | Fwd: 5’-ACCGGAAGTGTGGGATACTG-3’  Rev: 5’-GCTCACGGTTCCACTTCATT-3’  Fwd:5'-GGACCTGACCTGCCGTCTAG -3';  Rev:5'- TAGCCCAGGATGCCCTTGAG-3';  Fwd: 5′-CGAGCACTGCCCAACAACA-3'  Rev: 5'-CACGCCCACGGATCTGAA-3'  Fwd: 5′- CATAGTACTGGGTCTGGC-3';  Rev: 5'-CATACATCATTGGGTTACC-3'  Fwd:5′-GTGCCAGAAACCGTTGAATC-3'  Rev: 5'-TTGTGTTTCTCTGCGTCGTT-3' | 194  100  91  235  152  150 |
